# First Sounds of Emotion: Newborns' Sensitivity to Emotional Prosody and Its Relation With Maternal Depressive Traits

**DOI:** 10.1111/infa.70087

**Published:** 2026-04-23

**Authors:** Valentina Silvestri, Silvia Polver, Daniela Morniroli, Maria Lorella Giannì, Judit Gervain, Hermann Bulf

**Affiliations:** ^1^ Department of Psychology University of Milano‐Bicocca Milan Italy; ^2^ HUN‐REN Institute of Cognitive Neuroscience and Psychology Research Centre for Natural Sciences Budapest Hungary; ^3^ Laboratoire des Systèmes Perceptifs UMR CNRS Ecole Normale Supérieure PSL University Paris France; ^4^ Department of Developmental and Social Psychology University of Padua Padua Italy; ^5^ Department of Clinical Sciences and Community Health University of Milan Milan Italy; ^6^ Fondazione IRCCS Ca' Granda Ospedale Maggiore Policlinico NICU Milan Italy; ^7^ Padova Neuroscience Center University of Padua Padua Italy; ^8^ Integrative Neuroscience and Cognition Center (UMR8002) Université Paris Cité Paris France; ^9^ NeuroMI Milan Center for Neuroscience Milan Italy

**Keywords:** emotion processing, emotional prosody, maternal depressive traits, newborns, non‐nutritive sucking

## Abstract

The ability to perceive and respond to vocal emotional cues is critical for early social development, guiding infants' interactions with caregivers. Although newborns are believed to preferentially attend to positive prosody, the perceptual mechanisms underlying this response—as well as the degree to which these responses are language‐specific—remain poorly understood. This study examined newborns' sensitivity to emotional prosody above and beyond linguistic prosody marking different sentence types (i.e., interrogatives, imperatives, and declaratives) and its relation to maternal postnatal depressive traits. Forty‐three newborns were presented with interrogative, imperative, and declarative utterances spoken each with happy, neutral, and sad prosodies while their non‐nutritive sucking (NNS) behavior was measured and maternal depressive traits were assessed. Results revealed inhibited NNS behavior in response to sad and neutral utterances compared to happy ones (*ps* < 0.05), suggesting early differential responsiveness to positive vocal affect, independent of the linguistic prosodic contours of the sentence type. Moreover, reduced responsiveness to non‐positive prosodies was positively associated with maternal postnatal depressive traits, suggesting that variation in the early affective environment may shape newborns' auditory–emotional sensitivity. These findings highlight the interplay between biological predispositions and environmental influences in early emotional processing, emphasizing the role of early auditory experiences in shaping socio‐emotional development.

## 1 Introduction

The ability to perceive and respond to emotional cues is fundamental to early social development. Effectively monitoring others' emotional expressions enables individuals to anticipate and interpret their behavior. From the earliest stage of development, human infants exhibit remarkable sensitivity to a wide range of emotional signals, including vocalizations (Blasi et al. 2011), body language (Vuong and Geangu 2023; Zieber et al. 2014), and facial expressions (Addabbo et al. 2018; Farroni et al. 2007; Silvestri et al. 2024). Among these emotional cues, vocal prosody plays a pivotal role in early social interactions, conveying affective information through fluctuations in pitch, rhythm, and intensity (Latinus and Belin 2011; Lindquist et al. 2012). Vocal emotions thus serve as powerful signals, with their acoustic properties capable of evoking emotional responses in infants (Fernald 1993).

Emotional prosody is especially relevant as the auditory system becomes functional early in development, between the 24th–28th week of gestation. As the intrauterine environment attenuates sounds functioning as natural low‐pass filters (Griffiths et al. 1994; for a review, see Nallet and Gervain 2021), the fetus predominantly receives a range of frequencies in which suprasegmental, that is prosodic information such as the melody and rhythm of speech, are largely preserved, while segmental and thus lexical information is suppressed (Querleu et al. 1988).

Since prosody constitutes fetuses' earliest experience with language, at birth, infants already exhibit a heightened sensitivity not only to basic prosodic cues but also to utterance‐level intonational contours, representing the highest tier of the prosodic hierarchy (Abboub et al. 2016; Gervain 2018; Martinez‐Alvarez et al. 2023; Nazzi et al. 1998). In the current study, we, therefore, seek to examine whether newborn infants are sensitive to emotional prosody carried by utterances above and beyond linguistic prosody indicating sentence type, such as interrogatives, imperatives or declaratives.

In early interactions, caregivers use vocal prosody to express emotions and regulate infants' behavior in different situations (Vaish and Striano 2004). Although few studies have directly examined emotion‐specific responses to vocal prosodic cues in the early postnatal period, converging behavioral and electrophysiological evidence indicates that newborns are sensitive to vocal emotions from birth, displaying a pronounced preference for positive vocal expressions (Hou et al. 2024; Mastropieri and Turkewitz 1999; D. Zhang et al. 2019), with the exception of a few studies reporting heightened sensitivity to negatively valenced prosody (Cheng et al. 2012; D. Zhang et al. 2017).

More specifically, Mastropieri and Turkewitz (1999) demonstrated that newborns exposed to their native language exhibit increased sensitivity and preference to happy prosody relative to angry, sad, or neutral intonations. More recently, neurophysiological findings have reinforced the existing behavioral evidence, showing that sensitivity to emotional prosody emerges in preterm newborns as early as the 37th week of gestation (Hou et al. 2024). At this stage, a neural mismatch response—an early precursor to adult mismatch negativity—is triggered by positive vocal emotions and becomes further refined during the first year of life (Hou et al. 2024; Zhao et al. 2019). In contrast, neurophysiological evidence (Cheng et al. 2012; D. Zhang et al. 2017) revealed enhanced responses to fearful prosody relative to happy or neutral tones, highlighting the prioritization of negative emotional processing from birth, an evolutionary‐driven urgency in identifying potential threats (D. Zhang et al. 2017). Extending beyond the neonatal period, research on both infants (Grossmann et al. 2005; Zhao et al. 2021) and adults (Ito et al. 1998) supports the idea that the efficient decoding of vocal emotional cues is evolutionarily advantageous, facilitating rapid responses to potential dangers.

These findings might appear contradictory in highlighting both a preference for positive prosody (Mastropieri and Turkewitz 1999; Hou et al. 2024) and a responsiveness to negative vocal cues (Cheng et al. 2012; D. Zhang et al. 2017). However, this apparent inconsistency can be better understood in light of a dual‐sensitivity model, in which the early sensitivity to emotional prosody reflects an evolutionary strategy designed to elicit both caregiving responses (Lohaus et al. 2001) and to rapidly detect potential danger (Dykas and Cassidy 2011). Positive vocal cues signal safety and promote social bonding, whereas negative prosody serves as an alarm, drawing attention to possible dangers (Vaish et al. 2008; Vaish and Striano 2004). Two complementary mechanisms may underlie this dual sensitivity: an experience‐dependent tuning to the emotional cues prevalent in the social environment, which are often positively valenced, and an ontogenetic predisposition to swiftly recognize and respond to threatening cues (Silvestri et al. 2024). Newborns' immediate postnatal preferences for positive affective signals support the idea that sensitivity to positive cues may emerge rapidly through early social experience, in interaction with those predispositions (Farroni et al. 2007; Mastropieri and Turkewitz 1999). Overall, these findings underscore the critical interplay between innate neurobiological processes and postnatal experience in shaping the processing of emotional prosody.

While this dual mechanism offers a compelling framework, it remains unclear which factors modulate the relative salience of positive versus negative vocal cues, and how newborns actively process emotional prosody when it is embedded in naturalistic speech, in which emotional cues are embedded within complete sentences rather than isolated syllables (Zhao et al. 2019), vocalizations (Blasi et al. 2011; Zhao et al. 2021) or single words (Grossmann et al. 2005; Grossmann et al. 2010). Indeed, while prior research has largely relied on isolated stimuli, infants in real‐world settings are exposed to full utterances from which emotional prosody must be extracted and separated from linguistic prosody.

Furthermore, prior research predominantly focused on neurophysiological mechanisms (Grossmann et al. 2005; Hou et al. 2024; Zhao et al. 2021) underlying neural sensitivity to vocal affect. While these studies offer important insights into the neural correlates of prosody processing, little is known about how newborns overtly and actively process emotional speech in a naturalistic manner. Given the critical role of early vocal responsiveness in shaping infant‐caregiver interactions, it is important to identify behavioral markers of preference and arousal at birth.

To address these knowledge gaps, our study investigates newborns' preference for emotional speech, varying both prosodic affect (happy, sad, neutral) and linguistic structure (declarative, imperative, interrogative). By employing non‐nutritive sucking (NNS) as a proxy for preference, we aim to capture the early mechanisms underlying emotional prosodic processing. NNS provides a well‐established behavioral index of neonatal perceptual preference, leveraging the innate rhythmic sucking reflex to track sensory‐driven modulations in arousal and discrimination (Barlow and Estep 2006; Wolff 1968). This method has been extensively used to assess early perceptual sensitivities across modalities, including auditory processing (Byers‐Heinlein et al. 2010; Christophe et al. 2001; Moon et al. 2013). Here, we employ NNS to examine newborns' responsiveness to emotional prosody, capturing shifts in sucking activity in response to happy, sad, and neutral vocal stimuli compared to an initial silent baseline. Crucially, we also investigated whether this sensitivity to emotional prosody is modulated by the linguistic structure of the utterances by varying sentence types marked by their natural linguistic prosody (i.e., declarative, imperative, and interrogative). Indeed, this manipulation allowed us to examine whether newborns are differentially sensitive to emotional versus linguistic cues. Thus, by including both dimensions, we aimed to assess whether emotional content would emerge consistently across different sentence types as an overarching cue in the development of speech perception (Soderstrom et al. 2017).

This vocal sensitivity does not develop in isolation; rather, it unfolds within the infant's unique social environment, where interactions with primary caregivers, especially the mother, play a pivotal role in shaping communicative and socio‐emotional development. Maternal behaviors, such as contingent responsiveness and emotional availability, are known to facilitate infants' language acquisition and emotional regulation (Leigh et al., 2011; Stein et al. 2008). Conversely, maternal depressive traits may negatively impact the affective quality of caregiver–infant interactions. Specifically, maternal affective states have been shown to shape both the quality of infant‐directed speech and the infant's ability to process emotional cues (Aoyagi et al. 2019; Huffmeijer et al. 2020; Scheiber et al. 2022). Depressive traits in mothers can reduce the prosodic richness and the emotional expressiveness of their speech, potentially impacting how newborns attend to and discriminate vocal emotions. Given this, an important question is whether maternal depressive traits modulate neonatal sensitivity to emotional prosody from the earliest days of life. In the present study, we therefore assessed maternal postpartum mood (Cox et al. 1987) and its influence on newborns' preference for happy, neutral, and sad sentences.

## 2 Materials and Methods

### 2.1 Participants

A total of 43 healthy, full‐term newborns (20 females; mean age = 50 h, range = 18–78 h; mean weight at birth = 3114 gr, range = 2395–4030 gr) completed the study. An additional 8 infants were recruited and tested but were later excluded due to drowsiness/fussiness (*N* = 6), technical program errors (*N* = 1), or refusal of the pacifier (*N* = 1). The sample size was predetermined using a Monte Carlo simulation via the SIMR package in R (Green and Macleod 2016). We conducted an a priori power analysis based on the effect size of a pilot sample (*N* = 8), and after 1000 simulations, a sample size of 40 participants was considered sufficient to achieve a power of 93.9%.

Newborns were recruited from the Neonatology and and NICU Unit at the Fondazione IRCCS Ca’ Granda Ospedale Maggiore Policlinico. They all met the inclusion criteria of gestational age > 37 weeks and an Apgar score of at least 9 at 5 min, being exclusively exposed to Italian in utero, and all were tested approximately 1 hour before feeding when they were in an awake and alert state (i.e., showing standard behavioral indicators such as open eyes, spontaneous motor activity, and an active sucking reflex). They all successfully completed the routine newborn auditory screening performed in the maternity ward, came from middle‐class families and their parents self‐reported as White. Parental written informed consent was obtained before testing began. The protocol was carried out under the ethical standards of the Declaration of Helsinki (BMJ 1991; 302: 1194), and approved by the Ethics Committee of Milano Area 2 (ID: 694; Approval N. 952_2021).

### 2.2 Stimuli

One hundred twenty‐nine distinct sentences were created, categorized into three linguistic structures: 48 declaratives, 46 imperatives, and 35 interrogatives. The meanings of the sentences were chosen such that they allowed for all three emotions used: happy, sad and neutral, for example “Come here!”. Each sentence was recorded using the natural Italian intonational contour appropriate to its sentence type (declarative, interrogative, or imperative). Each utterance was recorded in three emotional prosodies by three different female native speakers of Italian. The final set of stimuli thus included 1161 utterances. Stimuli were designed to represent a broad range of linguistic patterns and to better approximate the linguistic environment newborns experience in daily interactions. The use of female voices was chosen for ecological validity, as infants in early development are predominantly exposed to maternal speech (Decasper and Fifer 1980; Cristia 2013).

To control for the contribution of lexical content and isolate emotional prosody, we conducted two preliminary validations: the first assessed the semantic neutrality of written sentences, and the second evaluated the perceived emotional tone of the corresponding audio recordings.

To ensure neutrality at the semantic level, a preliminary validation study was conducted on the written versions of all the candidate sentences. A total of 204 sentences, varying in length, were evaluated by 94 adult participants using an online survey through Qualtrics (Qualtrics, Provo, Utah, USA, https://www.qualtrics.com). Participants rated each sentence for valence on a 7‐point Likert scale (1 = very negative, 7 = very positive; Huffmeijer et al. 2020). Sentences that were judged to be excessively positive or negative were excluded (i.e., selection of those utterances that had an average rating larger than 3 and smaller than 5, and for which the extreme scores of 1 and 7 were not selected by any rater, as in Huffmeijer et al. 2020), leaving a final set of 129 semantically neutral sentences (48 declaratives, length range = 2–4 words; 46 imperatives, length range = 1–3 words; 35 interrogatives, length range = 2–4 words) as the proportion of excluded items differed across sentence types.

Three trained female speakers, that is professional actresses, were recruited to produce the final recordings, each performing all sentences in the three emotional prosodic conditions, happy, sad, and neutral. Multiple speakers were included to introduce natural acoustic variability, as all infants heard all three speakers in each condition. Recordings were conducted in a sound‐attenuated room using Audacity software, ensuring high‐quality audio capture. Post‐recording processing involved trimming silences at the beginning and end of each utterance, followed by acoustic analysis in Praat to extract key prosodic features, including mean duration and pitch. Both the mean duration and pitch were entered into two linear mixed‐effects models with Emotional prosody (happy, neutral, sad) as a fixed factor and random intercepts for Sentence and Actress Identity. The analysis on the mean pitch revealed a significant main effect of emotional prosody, *F* (2,1028) = 1398.3, *p* < 0.001, *R* ^2^ = 0.64, with higher pitch values for happy utterances (*M* = 276, SD = 27.9), lower values for neutral ones (*M* = 184, SD = 14.7), and sad utterances falling in between (*M* = 199, SD = 22.5), all *ps* < 0.001. For sentence duration, the analysis revealed a significant main effect of emotional prosody, *F* (2,1028) = 180.75, *p* < 0.001, *R* ^2^ = 0.08, with longer duration for sad utterances (*M* = 0.988, SD = 0.192) compared to both happy (*M* = 0.859, SD = 0.163), *p* < 0.001, and neutral ones (*M* = 0.847, SD = 0.188), *p* < 0.001, which did not differ significantly from each other, *p* = 0.11. These variations reflect characteristic temporal and spectral features of natural emotional expressions (Kamiloğlu et al. 2020; Vroomen et al. 1993).

To validate the emotional valence of the recordings, an online study was conducted using Labvanced (https://www.labvanced.com/). Participants were instructed to rate how happy, neutral, or sad each sentence sounded, focusing on intonation rather than semantic content. Given the large number of sentences, three subgroups of 60‐60‐55 items were selected for validation from three different groups of participants. Participants were instructed to rate how happy, neutral, or sad each sentence sounded. A total of 39 adult listeners (Group 1: *N* = 13; Group 2: *N* = 15; Group 3: *N* = 11) completed the validation. The mean endorsement rates for correctly identifying the intended prosody (Happy: 97.1%; Neutral: 95.4%; Sad: 94.6%) were analyzed using a generalized linear mixed‐effects logistic model. Emotional category (happy, neutral, sad) and Group were included as fixed effects, while random intercepts were specified for participant and stimulus item. The analysis revealed no significant main effects of Emotion or Group, nor their interaction (all *ps* > 0.10), indicating that all three emotions were similarly recognized by native speakers as intended.

Then all the stimuli were matched in intensity (70 dB) using Praat (v6.1.37). To preserve the ecological validity of the stimuli and retain natural emotional cues, we limited acoustic manipulations to intensity normalization.

### 2.3 Apparatus and Procedure

Approximately 1 hour before their scheduled feeding time and only if in an awake and alert state, parents brought their infants to the Neonatology Unit laboratory, a quiet testing room where background noise is minimized. This timing was chosen to maximize attentiveness, as neonatal reflexes, including sucking, are closely tied to alertness. Each newborn was seated on an experimenter's lap in a semi‐inclined position. To start the experimental procedure, the experimenter gently stroked the infant's cheek to trigger the sucking reflex and introduced a custom‐built pacifier, equipped with a pressure transducer connected to a computer for real‐time signal acquisition in the newborns' mouth (see NNS Recording and Processing section for details). Once the pacifier was in place, data recording commenced. Each pacifier was used only once and discarded after the session. To minimize potential external influences, the experimenter holding the infant refrained from wearing perfume or scented personal care products and avoided eye contact or interaction throughout the session. A second researcher was responsible for controlling the data acquisition system and ensuring proper signal recording. One or both parents remained in the room, positioned outside the infant's visual field, but could only interact with the infant if signs of distress emerged, in which case the session was halted. The room's lighting was dimmed for the experiment's duration.

Auditory stimuli were presented at 70 dB via free‐field speakers positioned behind the infant's head, with sound levels calibrated before each session. The study began with an initial 60‐s silent baseline period to measure spontaneous sucking behavior in the absence of stimulation. After the baseline period, newborns were presented with nine pseudo‐randomized blocks, each corresponding to a unique combination of emotional prosody (happy, sad, neutral) and sentence type (declarative, imperative, interrogative), resulting in the following conditions: Happy Declarative, Happy Imperative, Happy Interrogative, Neutral Declarative, Neutral Imperative, Neutral Interrogative, Sad Declarative, Sad Imperative, and Sad Interrogative. The order of the nine blocks was pseudorandomized across participants, with the constraint that neither the same emotional prosody nor the same sentence type could occur consecutively to prevent long runs of similar conditions. Each block lasted 60 s and was composed of 30 utterances, with a stimulus‐onset asynchrony of 2 s. Because utterance durations varied between 245 ms and 1810 ms, the inter‐stimulus interval (ISI) ranged from 1755 ms to 190 ms, depending on the length of the utterance. Stimuli within each block were randomized to avoid repetition of identical sentences or speaker identities within the same block. The total session lasted approximately 10 min for each newborn. Newborns contributed, on average, 9.79 valid trials (SD = 0.41) across the 10 conditions.

After the task administered to newborns, maternal depressive traits were assessed using the Edinburgh Postnatal Depression Scale (EPDS, Cox et al. 1987), a widely validated screening instrument for detecting symptoms of perinatal depression. The EPDS consists of 10 self‐report items, each rated on a 4‐point Likert scale ranging from 0 (absence of symptoms) to 3 (high symptom severity), with total scores reflecting the intensity of depressive symptomatology. Scores range from 0 to 30, with higher scores indicating higher levels of depressed mood. A cutoff of 13 indicates a high probability of clinical depression. The scale captures a broad spectrum of emotional and behavioral symptoms experienced over the previous week, including low mood (e.g., “I have been so unhappy that I have been crying”), self‐blame (e.g., “I have blamed myself unnecessarily when things went wrong”), anxiety (e.g., “I have been anxious or worried for no good reason”), sleep disturbances (e.g., “I have been so unhappy that I have had difficulty sleeping”), and self‐harm ideation (e.g., “The thought of harming myself has occurred to me”). Out of the 43 participants in the study, 35 mothers (81.4%) completed the questionnaire. Eight mothers did not complete the EPDS because they were no longer available at the time of questionnaire administration (i.e., due to early hospital discharge, *N* = 6, or scheduling constraints, *N* = 2).

### 2.4 NNS Recording and Processing

Newborns' responses were assessed using the non‐nutritive sucking (NNS) method, monitored via a pacifier equipped with a pressure transducer connected to a computer for real‐time signal acquisition. The raw signal was processed using MATLAB (Mathworks, Natick, MA; ver. 2022b). Baseline correction was applied by subtracting the resting pressure value to remove artifacts related to pacifier contact. The signal was then down‐sampled to 100 Hz to optimize computational efficiency while preserving the temporal structure of sucking patterns, which typically occur at low frequencies (2 Hz). A band‐pass filter (0.5–20 Hz) was implemented to eliminate low‐frequency artifacts from jaw and tongue movements unrelated to sucking, as well as high‐frequency noise. Lastly, a smoothing procedure was applied to enhance signal quality and minimize residual noise (Barlow et al. 2014, 2023). Sucking cycles per trial were identified using MATLAB's *findpeaks* function. A cycle was defined as an event where pressure exceeded 1.5 mbar, as in previous studies (Barlow et al. 2014, 2023; Liao et al. 2019; Silvestri et al. 2025) to reliably identify true sucking events from non‐sucking oral movements. The number of cycles per trial was extracted for each newborn and for each completed trial.

### 2.5 Statistical Models

Newborns were presented with a silent period of no stimulation (i.e., baseline condition) followed by the nine stimulation conditions (i.e., Happy Declarative, Happy Imperative, Happy Interrogative, Neutral Declarative, Neutral Imperative, Neutral Interrogative, Sad Declarative, Sad Imperative, and Sad Interrogative).

To investigate newborns' sensitivity to emotional speech (i.e., any of the three emotional conditions compared to the initial silent baseline), we ran a linear mixed effects model (LMM) on the dependent variable number of cycles/trial (NNS cycle/trials) with Conditions (baseline, neutral, happy and sad) as fixed factors and Participants as a random factor with a random intercept.

To investigate newborns' differential sensitivity to emotional content (i.e., neutral, happy, sad) based on sentence types (i.e., declarative, interrogative, imperative) we ran a linear mixed effects model (LMM) on the dependent variable NNS cycles/trial. We included Emotional condition and Sentence types as fixed factors and Participants as a random factor with a random intercept. As in previous studies with newborns (Arioli et al. 2025; Silvestri et al. 2025), only random intercepts were included because each infant contributed with only one value per condition, making slope estimation statistically unsupported and prone to convergence failures (Barr et al. 2013).

We used the lme4 package in R (version 4.2.3), with Satterthwaite approximations for degrees of freedom (usually suitable for datasets with smaller numbers of subjects and observations). Residual diagnostics indicated that the assumptions of normality and homoscedasticity were reasonably met. Standardized residuals approximated a normal distribution, as confirmed by visual inspection of Q–Q plots.

Lastly, to explore whether maternal postnatal depressive traits impact newborns' responses to emotional prosody, we ran Pearson correlations between the sucking behavior (i.e., NNS cycles/trial) in the baseline, neutral, happy, and sad conditions and depressive traits measured with the EPDS. Pearson's correlations were calculated using the function “corr.test” implemented in the R‐library *psych* (Revelle and Revelle 2015). Given the number of comparisons, their significance level was adjusted using False Discovery Rate (Benjamini and Hochberg 1995).

## 3 Results

Descriptive statistics for NNS cycles/trial and maternal EPDS scores are reported in Table 1. In the model comparing newborns' sucking behavior in the Emotional conditions (neutral, happy, sad, baseline), we found a main effect of Emotional condition, *F* (3, 375) = 3.66, *p* = 0.013, *R* ^2^ = 0.01, with newborns sucking less for neutral utterances, (*M* = 35.3, SE = 2.5), *t* (375.2) = −2.10, 95% CI [−10.37, −0.35], *p* = 0.036, *β* = −0.27, and sad ones, (*M* = 35, SE = 2.48), t (375.1) = −2.24, 95% CI [−10.76, −0.71], *p* = 0.026, *β* = −0.29, compared to the initial baseline (*M* = 40.7, SE = 3.07). No differences were found between happy utterances (*M* = 39.6, SE = 2.48) and the initial baseline period, *t* (375.1) = −0.49, 95% CI [−6.17, 3.88], *p* = 0.65.

**TABLE 1:** Descriptive statistics (mean, SD, maximum) for newborns' sucking behavior (NNS cycles/trial) across emotional prosodies, and maternal EPDS scores.

	Mean	SD	Maximum
NNS cycles/trial happy	39.56	18.70	82
NNS cycles/trial neutral	35.92	20.08	79
NNS cycles/trial sad	34.94	21.03	80
EPDS (maternal)	6.54	3.27	12

**FIGURE 1:**
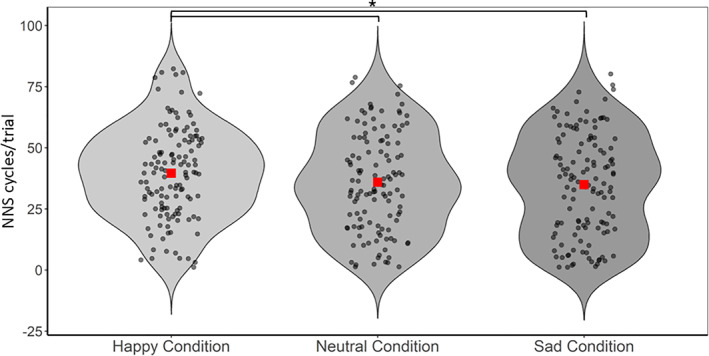
NNS across happy, neutral, and sad conditions. Single data points of each subject for NNS cycles/trial for happy, neutral, and sad conditions. The red square represents the group mean. *p < 0.05. **p < 0.005. ***p < 0.001.

The second model exploring newborns' sensitivity to Emotional content (neutral, happy, and sad) and Sentence types (declarative, interrogative, imperative) revealed a main effect of Emotional content, *F* (2, 327) = 3.92, *p* = 0.021, *R* ^2^ = 0.0132, with newborns sucking more for happy utterances (*M* = 39.6, SE = 2.47) than for neutral (*M* = 35.3, SE = 2.49), *t* (327.4) = −2.30, 95% CI [−7.86, −0.61], *p* = 0.022, *β* = −0.14, and sad utterances (*M* = 35, SE = 2.47), *t* (327.1) = −2.52, 95% CI [−8.16, −1.01], *p* = 0.012, *β* = −0.15, see Figure 1. No main effect of Sentence type, nor an interaction between Sentence type and Emotional content, was found.

**FIGURE 2:**
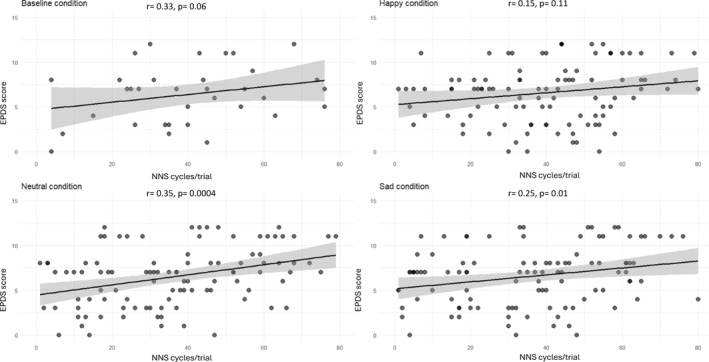
Correlations between EPDS score and NNS cycles/trial. Representation of the correlations in baseline (top left), happy (top right), neutral (bottom left), and sad (bottom right) conditions between EPDS score and NNS cycles/trial.

Lastly, to unravel the association between maternal depressive traits through the EPDS score and the NNS cycles/trial, we run correlations separately for the baseline, neutral, happy, and sad conditions (*N* = 35). After False Discovery Rate correction across the four tests, we found significant correlations between the EPDS score and the NNS cycles/trial in the neutral (*r* = 0.35, 95% CI [0.16, 0.51], *p* = 0.0004, *p*FDR = 0.001) and sad conditions (*r* = 0.25, 95% CI [0.06, 0.42], *p* = 0.01, *p*FDR = 0.017) (Figure 2). No significant correlations were found in the baseline and happy conditions (all *ps* > 0.06).

## 4 Discussion

The present study provides novel behavioral evidence that newborns actively differentiate emotional prosody embedded in naturalistic speech. Newborns first experienced a silent baseline period with no auditory stimulation, followed by nine experimental conditions comprising emotional utterances that varied in both prosodic affect (happy, neutral, sad) and sentence type (declarative, imperative, interrogative). Newborns' responses were measured using the non‐nutritive sucking (NNS) paradigm, which capitalizes on the innate, rhythmic sucking reflex—a robust and universal motor behavior present from birth in healthy infants (Barlow and Estep 2006; Zimmerman and Foran 2017).

Overall, we found that compared to a silent baseline, newborns significantly reduced their sucking behavior when exposed to neutral and sad utterances, suggesting a withdrawal or freezing response to these cues. Moreover, when directly comparing emotional conditions based on the sentence's linguistic type, happy utterances elicited significantly more sucking than both neutral and sad ones, suggesting enhanced engagement and preference. Notably, this effect was independent of sentence type, as no main effect or interaction involving the syntactic structure of the utterances (declarative, imperative, or interrogative) emerged, suggesting that emotional prosody may exert a stronger influence than linguistic prosody at birth. Although evidence exists that infants are capable of discriminating sentence‐level prosodic structures (Geffen and Mintz 2011), the present findings do not allow us to determine whether newborns processed linguistic prosody, as the emotional prosody may have overshadowed or overridden linguistic prosodic cues.

These results align with prior evidence suggesting that salient auditory stimuli can modulate sucking behavior by triggering responses that compete with the spontaneous sucking activity (Moon et al. 2013; Zimmerman and Foran 2017). Specifically, the reduction in sucking observed for neutral and sad utterances compared to baseline may reflect a freeze or withdrawal response, in line with studies showing that cognitive load or novel auditory features can inhibit motor behavior, reducing the frequency and duration of NNS in newborns (Moon et al. 2013; Valenza et al. 1994; Zimmerman and Foran 2017). In contrast, the lack of inhibition in response to happy utterances relative to baseline suggests a qualitatively distinct response, potentially driven by the prosodic patterns most frequently encountered in utero. Indeed, this dual pattern is consistent with evidence in literature concurrently showing that novel or cognitively demanding stimuli often elicit a motor inhibition response in NNS behavior (Moon et al. 2013; Zimmerman and Foran 2017), while familiar or preferred stimuli may enhance behavioral engagement (e.g., Decasper and Fifer 1980; Zimmerman and DeSousa 2018). Our results contribute to this debate by showing that neutral and sad utterances may be perceived as less familiar based on newborns’ early auditory experience, thus less encountered in the prenatal auditory environment which is typically rich in positive, melodic, and emotionally expressive speech (Arenillas‐Alcón et al. 2023; Vogelsang et al. 2023). In line with this, the absence of inhibition in NNS for happy utterances may reflect a facilitation effect associated with greater familiarity or innate preference for positive affective prosody, likely promoting approach behaviors and engagement. These findings help bridge two perspectives on non‐nutritive sucking: one that emphasizes inhibition in response to novel stimuli (Moon et al. 2013; Zimmerman and Foran 2017; Valenza et al. 1994), and another that interprets increased sucking as an indicator of preference or familiarity (Decasper and Fifer 1980; Zimmerman and DeSousa 2018). The current results demonstrate a valence‐specific modulation of newborns' motor behavior in response to emotional prosody—marked by inhibition for novel or neutral cues and facilitation for positive, familiar ones.

In addition, our findings contribute to the growing body of literature suggesting that sensitivity to emotional vocal cues is present from birth and may reflect an evolutionarily tuned mechanism for navigating early social environments. This sensitivity may be shaped by both prenatal exposure to maternal speech and postnatal interactions, where positive vocal cues signal safety and promote social bonding, while ambiguous (neutral) or negative (sad) tones prompt alertness to possible threats. Indeed, the absence of freezing response for happy prosody observed in the present study aligns with previous research showing newborns' preference for positive affective vocalizations (Mastropieri and Turkewitz 1999; Hou et al. 2024). Conversely, the reduced sucking rates for sad and neutral utterances mirror neurophysiological evidence suggesting heightened cortical responses to negative emotional prosody, facilitating responsiveness to potential dangers (Cheng et al. 2012; D. Zhang et al. 2017). Interestingly, in our study, sad and neutral utterances elicited similarly reduced sucking responses, suggesting that newborns may process both types of prosody as emotionally non‐rewarding or ambiguous. One possible explanation is that neutral prosody may be perceived as affectively ambiguous and, as a result, interpreted more like negative affect than positive. This interpretation is supported by findings from face perception literature, where neutral facial expressions are often judged as ambiguous or slightly negative, particularly during childhood (Tottenham et al. 2013) and where comparable activation of the amygdala for neutral and negative emotions was found (Lobaugh et al. 2006; Thomas et al. 2001). A similar mechanism may operate in the auditory domain, leading newborns to respond to neutral prosody with increased caution or reduced approach behavior (Blasi et al. 2011; Wambacq and Jerger 2004). It is also worth noting that sad and neutral prosodies differed in pitch in our stimuli, yet elicited virtually identical behavioral responses. This convergence makes it unlikely that the observed pattern is driven by acoustic salience or pitch differences alone. It should also be considered that emotional prosodies naturally vary in fundamental frequency, and equating them artificially would compromise ecological validity. Moreover, recent evidence shows that newborns discriminate positive emotional prosody, but not acoustically matched control sounds that share the same F0 contours (Hou et al. 2024), indicating that low‐level acoustic properties alone cannot account for neonatal sensitivity to emotional prosody.

From this perspective, our behavioral results support the idea of a dual sensitivity model for positive and negative vocal cues (Silvestri et al. 2024), proposing the coexistence of innate orienting mechanisms toward salient positive stimuli and early avoidance responses to negative cues, in which distinct strategies are engaged depending on the valence and familiarity of vocal input. Within this model, positive and negative emotional cues are both salient, and engage complementary processing mechanisms—one oriented toward social approach and bonding, and the other toward risk detection and caution. Such a system would confer evolutionary advantages by enabling newborns to flexibly adapt their behavior in response to the emotional context of the caregiver's voice. Notably, this study extends the framework commonly used in face perception research (Silvestri et al. 2024) by demonstrating that newborns not only detect emotional prosody but also modulate their behavioral responses based on its valence—a valence‐based modulation that can be measured using the non‐nutritive sucking method. Nonetheless, further investigation is needed to determine whether the reduced NNS in response to negative prosody reflects attentional disengagement, affective withdrawal, or early emotion regulation strategies.

Such valence‐driven responses may be further shaped by individual differences in prenatal experience, particularly the affective environment provided by the caregiver during pregnancy. Our results revealed significant positive correlations between EPDS scores and the number of cycles per trial for both neutral and sad utterances, but not for the happy condition, indicating that newborns' behavioral sensitivity to emotional prosody may already be modulated by early environmental factors. Critically, the association between maternal EPDS scores and infants' NNS in response to neutral and sad prosody—but not to happy prosody—may reflect underlying mechanisms shaped by prenatal auditory experience and early caregiving expectations. One possible interpretation is that newborns whose mothers showed higher postnatal depressive traits may have been more frequently exposed in utero to prosodic patterns characterized by reduced emotional expressivity or negative tone (Bettes 1988; Kaplan et al. 2001; Lam‐Cassettari and Kohlhoff 2020). Indeed, maternal depressive traits are known to alter the affective tone and prosodic richness of their speech (Aoyagi et al. 2019; Scheiber et al. 2022), potentially shaping the fetus's expectations about vocal emotional cues. Consequently, neutral and sad utterances may have been perceived as more familiar and less salient by newborns, resulting in reduced freezing and increased sucking behavior. Although higher sucking rates in newborns of mothers with elevated EPDS scores may resemble a preference for neutral or sad prosody, this interpretation should be treated with caution. The pattern likely reflects an attenuation of the typical behavioral inhibition observed for non‐positive affect (Schaadt et al. 2022). Such attenuation may involve a modulation in prosodic tuning shaped by exposure to reduced emotional expressivity. An alternative, yet complementary, speculative explanation draws from models of infant social engagement, which conceptualize the newborn not as a passive receiver but as an active participant in early communicative exchanges (Gros‐Louis et al. 2006; Renzi et al. 2017; V. H. Zhang et al. 2024). In this view, infants exposed to more affectively flat or negative speech might suck more in response to sad or neutral prosody as a way of prompting interaction or emotional feedback from the caregiver, a way to re‐establish reciprocity (Mesman et al. 2010; Tronick et al. 1978). Such dynamics suggest that even in the earliest days of life, newborns are sensitive to subtle contingencies in the emotional quality of vocal input and may adjust their processing strategies based on prior experience and expectations about caregiver responsiveness.

These results highlight the role of early caregiving context in shaping newborns' auditory preferences, suggesting that even subclinical variations in maternal mood (all mothers in our sample scored below the clinical EPDS cut‐off of 13) may influence how infants respond to emotional speech, potentially through mechanisms such as reduced exposure to rich prosodic cues or altered maternal speech affectivity. It is important to note, however, that these associations were modest in magnitude, and therefore should be interpreted as subtle modulations of newborns' prosodic responsiveness rather than strong predictive effects. Also, although postnatal EPDS scores are correlated with antenatal depressive symptoms (e.g., Heron et al. 2004), we did not directly determine whether the observed associations with newborns' prosodic sensitivity reflect exposure to maternal affective tone during pregnancy. Therefore, any reference to prenatal exposure should be viewed as a cautious interpretation, since it was not directly measured in the present study. Future studies should include antenatal measures of maternal mood to more precisely capture the prenatal emotional environment and disentangle its effects from those emerging after birth. In addition, the demographic homogeneity of our sample, composed exclusively of newborns from White, middle‐class families, and the absence of parental demographic information (e.g., age, education, SES), limits the generalizability of the present findings. Moreover, we did not record other perinatal factors known to influence NNS behavior, such as exposure to the maternal voice, feeding type (breastfeeding vs. bottle‐feeding), or delivery mode, which should be considered in future research. Indeed, future work should therefore examine more socio‐economically diverse populations to better assess the robustness and specificity of these effects. Also, future studies could expand on these findings by including clinical samples in longitudinal designs to better understand the interplay between prosody perception, maternal affect, and developmental outcomes. Thus, further evidence is needed to understand how infants' sensitivity to prosodic emotion processing affects subsequent emotional development, vocabulary acquisition, and other aspects of socio‐linguistic development (Lindquist and Gendron 2013; Nencheva et al. 2023; Nencheva, Tamir, et al. 2023).

Overall, by using ecological stimuli and a sensitive behavioral measure, this study offers a valuable window into the architecture of socio‐emotional processing at birth, showing that the valence‐based modulation of newborn behavior is not only an ontogenetic mechanism but also shaped by the caregivers' prenatal emotional affective status. These findings offer new insights into the behavioral underpinnings of prosody processing in early development, underscoring the relevance of both innate predispositions and postnatal environmental factors.

## Author Contributions

**Valentina Silvestri:** conceptualization, methodology, formal analysis, investigation, visualization, writing – original draft, writing – review and editing. **Silvia Polver:** conceptualization, methodology, writing – review and editing. **Daniela Morniroli:** investigation, writing – review and editing. **Maria Lorella Giannì:** writing – review and editing, resources, supervision. **Judit Gervain:** conceptualization, methodology, writing – review and editing, supervision. **Hermann Bulf:** conceptualization, methodology, writing – review and editing, resources, supervision.

## Funding

This work was supported by a grant from the Italian Ministry of University and Research ‐ NextGenerationEU (PNRR M4.C2.I1.1‐ Avviso 104/2022, CUP H53D23004110006, Grant 2022‐NAZ‐ 0410) ‐ awarded to Hermann Bulf.

## Ethics Statement

The study was conducted in accordance with the Declaration of Helsinki and the protocol was approved by the Milano‐Bicocca Ethical and Scientific Committees.

## Consent

Parents gave informed consent for infants' participation.

## Conflicts of Interest

The authors declare no conflicts of interest.

## Data Availability

The data that support the findings of this study are openly available in OSF at https://osf.io/6hnej/?view_only=ae912abbbcb44c53954389d7c9fcae84.
